# Transgenic *Arabidopsis thaliana* plants expressing bacterial γ-hexachlorocyclohexane dehydrochlorinase LinA

**DOI:** 10.1186/s12896-024-00867-0

**Published:** 2024-06-19

**Authors:** Wenhao Deng, Yoshinobu Takada, Yoshihiko Nanasato, Kouhei Kishida, Leonardo Stari, Yoshiyuki Ohtsubo, Yutaka Tabei, Masao Watanabe, Yuji Nagata

**Affiliations:** 1 Department of Molecular and Chemical Life Sciences, Graduate School of Life Sciences, Tohoku University, Sendai, 980-8577 Japan; 2 Forest Bio-Research Center, Forestry and Forest Products Research Institute (FFPRI), Forest Research and Management Organization (FRMO), 3809-1 Ishi, Juo, Hitachi, Ibaraki 319-1301 Japan; 3 Faculty of Food and Nutritional Sciences, Toyo University, 1-1-1 Izumino, Itakura-Machi, Ora-Gun, Gunma, 374-0193 Japan

**Keywords:** Transgenic plants, Phytoremediation, Organochlorine pesticides, POPs, γ-HCH dehydrochlorinase, *Arabidopsis thaliana*

## Abstract

**Background:**

γ-Hexachlorocyclohexane (γ-HCH), an organochlorine insecticide of anthropogenic origin, is a persistent organic pollutant (POP) that causes environmental pollution concerns worldwide. Although many γ-HCH-degrading bacterial strains are available, inoculating them directly into γ-HCH-contaminated soil is ineffective because of the low survival rate of the exogenous bacteria. Another strategy for the bioremediation of γ-HCH involves the use of transgenic plants expressing bacterial enzyme for γ-HCH degradation through phytoremediation.

**Results:**

We generated transgenic *Arabidopsis thaliana* expressing γ-HCH dehydrochlroninase LinA from bacterium *Sphingobium japonicum* strain UT26. Among the transgenic *Arabidopsis* T2 lines, we obtained one line (A5) that expressed and accumulated LinA well. The A5-derived T3 plants showed higher tolerance to γ-HCH than the non-transformant control plants, indicating that γ-HCH is toxic for *Arabidopsis thaliana* and that this effect is relieved by LinA expression. The crude extract of the A5 plants showed γ-HCH degradation activity, and metabolites of γ-HCH produced by the LinA reaction were detected in the assay solution, indicating that the A5 plants accumulated the active LinA protein. In some A5 lines, the whole plant absorbed and degraded more than 99% of γ-HCH (10 ppm) in the liquid medium within 36 h.

**Conclusion:**

The transgenic *Arabidopsis* expressing active LinA absorbed and degraded γ-HCH in the liquid medium, indicating the high potential of LinA-expressing transgenic plants for the phytoremediation of environmental γ-HCH. This study marks a crucial step toward the practical use of transgenic plants for the phytoremediation of POPs.

**Supplementary Information:**

The online version contains supplementary material available at 10.1186/s12896-024-00867-0.

## Background

γ-Hexachlorocyclohexane (γ-HCH), an organochlorine insecticide of anthropogenic origin, was used worldwide in the 1940s. Its release into the environment caused serious environmental problems due to its toxicity and long-term persistence in upland soils [1–3]. Although many countries have prohibited its use, γ-HCH remains in environments around the world [1], and γ-HCH and its byproducts, α-HCH and β-HCH, were listed as persistent organic pollutants (POPs) at the Stockholm Convention [3]. Thus, there is a global need for HCH removal and remediation actions.

Many bacterial strains, notably those from Sphingomonadaceae family, have been extensively studied at the molecular level for their ability to degrade γ-HCH and its isomers under aerobic conditions [2, 4, 5]. However, it was strongly suggested that bacterial activities in natural environments differ significantly from those under laboratory conditions [6–8]. We are now encountering problems with the practical use of bacteria in complex natural environments (*e.g*., bioremediation and biological control of plant diseases) since bacteria do not always perform the functions they exhibited under laboratory conditions [6, 9, 10]. Indeed, direct inoculation of bacteria into the soil can affect the viability of inoculated organisms due to factors such as temperature, humidity, pH, organic matter content, and biological predators and competitors [11].

Phytoremediation is another strategy to remove organic and inorganic contaminants from the environment [12]. The low cost of phytoremediation makes it suitable for the remediation of large areas of contaminated land. However, two problems present obstacles to the practical use of phytoremediation in soils contaminated with hydrophobic and recalcitrant compounds like γ-HCH: plants are generally unable to absorb large amounts of hydrophobic compounds, and they lack enzymes that degrade recalcitrant compounds such as γ-HCH.

*Cucurbita* species can take up a large number of POPs from soil [13–16]. It was reported that major latex-like proteins (MLPs) in their xylem sap are related to the efficient translocation of hydrophobic contaminants [17]. Therefore, we reasoned that generating transgenic *Cucurbita* species expressing γ-HCH-degrading enzymes might show promise as a new strategy for phytoremediation of γ-HCH. Our previous study produced transgenic hairy root cultures of *Cucurbita moschata* expressing bacterial γ-HCH dehydrochlorinase LinA [18]. In that study, we synthesized codon-optimized *linA* (*relinA*) (Accession No. LC006108), and further modified it to remove unwanted restriction enzyme recognition sites and codons that plant cells rarely use. The final *relinA* sequence comprised 74 (16%) modified base pairs (bp) that did not change the predicted amino acid sequence of LinA [18]. In addition, it was necessary to fuse an endoplasmic reticulum targeting signal peptide to LinA for stable accumulation in hairy roots. The resultant LinA-expressed transgenic hairy root cultures successfully degraded γ-HCH, indicating that LinA has strong potential for phytoremediation of environmental γ-HCH [18].

**Fig. 1:**
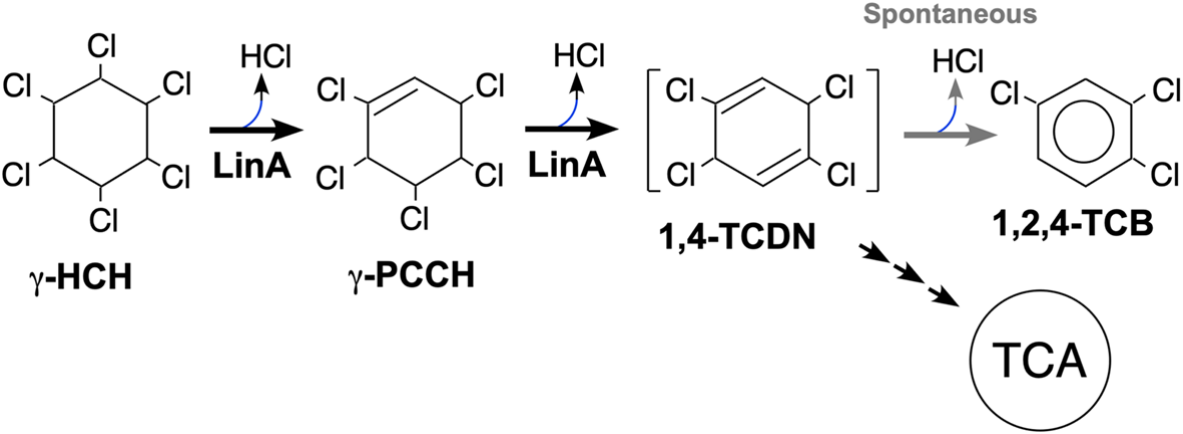
Reactions catalyzed by γ-HCH dehydrochlorinase LinA. LinA catalyzes the first two steps of the γ-HCH degradation in γ-HCH-degrading bacteria *Sphingobium japonicum* strain UT26 [19, 20]. LinA converts γ-HCH to 1,3,4,6-tetrachloro-1,4-cyclohexadiene (1,4-TCDN) via γ-pentachlorocyclohexene (γ-PCCH). 1,4-TCDN is further metabolized to TCA in UT26 [4]. 1,4-TCDN is hypothesized to undergo a nonenzymatic conversion to 1,2,4-trichlorobenzene (1,2,4-TCB) due to the inherent instability of its diene-type structure, leading to its transformation into the more stable aromatic ring structure of 1,2,4-TCB [21]

LinA is a unique dehydrochlorinase with no significant homologous sequence [19, 20]. LinA was originally identified as an enzyme that catalyzes the first two steps of dehydrochlorination from γ-HCH to 1,3,4,6-tetrachloro-1,4-cyclohexadiene (1,4-TCDN) via γ-pentachlorocyclohexene (γ-PCCH) (Fig. 1) in *Sphingobium japonicum* strain UT26 [19, 20]. This strain is one of the best characterized of the bacterial strains degrading γ-HCH and has been the focus of extensive analyses of the degradation pathway of γ-HCH [4]. 1,4-TCDN is hypothesized to undergo a nonenzymatic conversion to 1,2,4-trichlorobenzene (1,2,4-TCB) due to the inherent instability of its diene-type structure, leading to its transformation into the more stable aromatic ring structure of 1,2,4-TCB (Fig. 1) [21].

In addition to γ-HCH and γ-PCCH, LinA also catalyzes the dehydrochlorination of α-, δ-, and ε-HCHs and the corresponding PCCHs [20, 22, 23]. Dehydrochlorination by LinA occurs stereoselectively by acting specifically on a *trans* and diaxial pair of hydrogen and chlorine atoms [21, 24]. The overall three-dimensional structure of LinA predicted by computer modelling was verified by site-directed mutagenesis, and its D25, H73, and R129 residues were shown to form catalytically critical residues of LinA and to be essential for its activity [25]. Later, the crystal structure of LinA was experimentally solved, and it supported the proposed 1,2-anti dehydrochlorination of γ-HCH by LinA [26]. LinA did not dehydrochlorinate the other chlorinated compounds tested [20], indicating its narrow substrate specificity. Hexabromocyclododecanes were the only reported substrates of LinA besides HCH-related compounds [27–29]. In addition, we recently demonstrated that LinA could also convert another notorious synthetic organochlorine insecticide, 1,1,1-trichloro-2,2-bis(4-chlorophenyl)-ethane (DDT), which is also listed as a POP, to 1,1-dichloro-2,2-bis(4-chlorophenyl)-ethylene (DDE) [30], indicating the high potential of LinA for the bioremediation of POPs. LinA catalyzes the reaction without any cofactor, and this feature is an advantage for the heterologous expression of LinA [20].

In the previous study, we produced transgenic hairy root cultures of *C. moschata* expressing LinA [18]. For practical application, however, it is crucial to generate the whole transgenic plant expressing LinA. Thus, in this study we used the *Arabidopsis thaliana* plant, which grows fast and is easily modified genetically, to assess the possibility of using LinA-expressing transgenic plants for the phytoremediation of γ-HCH.

## Results

### Production of transgenic *Arabidopsis* into which the *relinA* gene was introduced

**Fig. 2:**
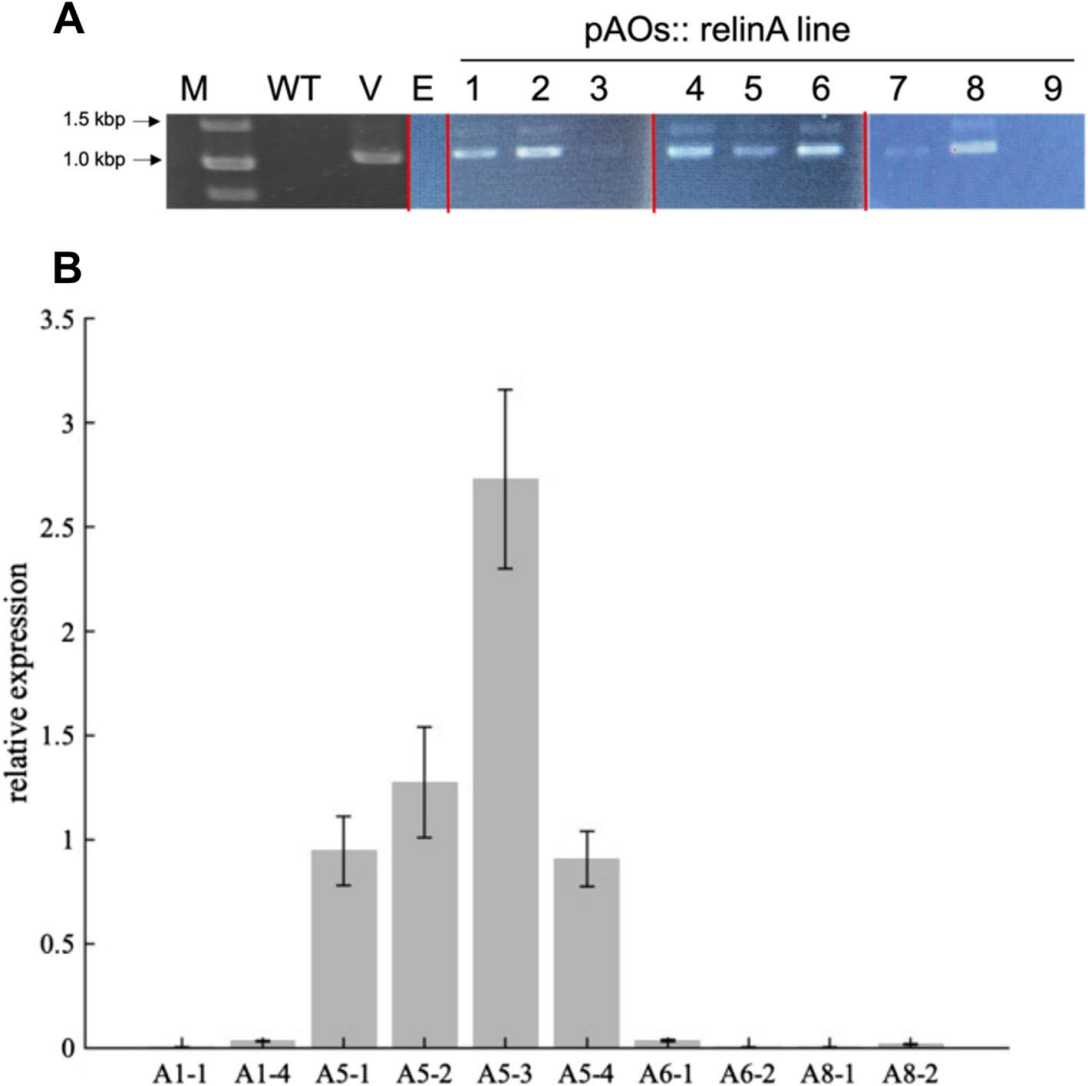
Production and expression analysis of transgenic *A. thaliana*. **A** PCR analysis of the genomic DNA of T2 *Arabidopsis* lines transformed with pAOs::relinA (lane 1 to 9 indicates T2 transgenic lines, A1 to A9, respectively). Theoretically, 1,089 bp DNA is amplified by the primer set for the *relinA* gene. M, 1-kb ladder of DNA size markers; WT, genomic DNA of wide type *Arabidopsis*; E, genomic DNA of *Arabidopsis* line transformed with control vector pIG-sGFP [32]; V, plasmid DNA of pAOs::relinA (positive control). The full uncropped gel images for this figure is provided as Figure S1. **B** RT-qPCR analysis of *relinA* gene transcription in T3 transgenic *Arabidopsis* lines. The relative expression level of *relinA* to that of the endogenous reference gene *ACTIN2* is shown. Error bars indicate the mean ± standard deviation (SD), *n* = 3 (biological replicates)

In the previous study, we generated hairy root cultures of *C. moschata* that express LinA fused to the ER-targeting signal peptide from the ascorbate oxidase of *Cucumis sativus* [31], named AOs hereafter [18]. Using pAOs::relinA, the same vector we used in the previous study, we transformed *A. thaliana*. PCR analysis confirmed the *relinA* gene's introduction, and six independent T2 lines (A1, A2, A4, A5, A6, and A8) of transgenic *A. thaliana* were obtained (Fig. 2A). We conducted subculture for the selected six T2 lines and retained 2 to 4 individual T3 plants that grew well for each line. We then conducted RT-qPCR analysis of 10 individual T3 lines for expression of *relinA* at the transcriptional level (Fig. 2B). Four A5-derived T3 lines (A5-1, A5-2, A5-3, and A5-4) showed apparent *relinA* expression at the transcriptional level. Of these, A5-3 expressed *relinA* at the highest level.

### Selection of transgenic *Arabidopsis* plants expressing LinA

**Fig. 3:**
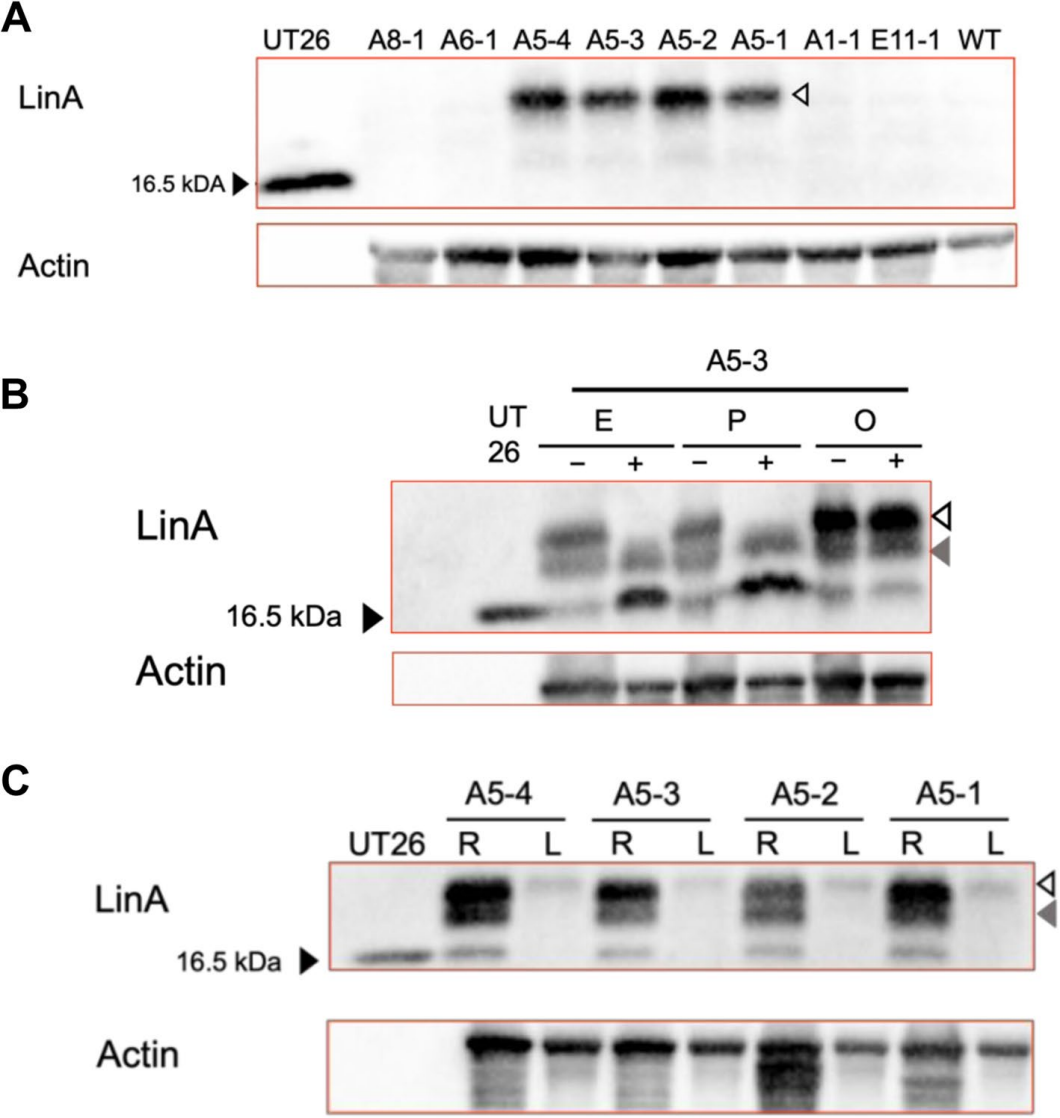
Analysis of LinA expressed in transgenic *Arabidopsis*. **A** Western blot analysis of protein extracts of T3 transgenic *Arabidopsis* lines transformed with pAOs::relinA (A8-1, A6-1, A5-4, A5-3, A5-2, A5-1, and A1-1) and with control empty vector (E11-1), wild type *Arabidopsis* (WT), and bacterial strain UT26 cells (UT26) was performed using an anti-LinA antibody. Actin was used as a loading control. Black and white triangles indicate native LinA expressed in UT26 cells and LinA expressed in transgenic *Arabidopsis*, respectively. **B** Analysis of the glycosylation of LinA expressed in transgenic *Arabidopsis* line. Protein extract of A5-3 line was treated with ( +) or without (-) glycopeptidases, Endo H (E), PNGase F (P), and *O*-glycosidase (O). Western blot analysis was performed using an anti-LinA antibody. Actin was used as a loading control. Black and white triangles indicate native LinA expressed in UT26 cells and *N*-glycosylated LinA expressed in transgenic *Arabidopsis*, respectively. Gray triangle indicates ca. 17-kDa protein expressed in transgenic *Arabidopsis* with an unknown modification*.* **C** Western blot analysis of protein extracts from root (R) and leaf (L) of transgenic *Arabidopsis* was performed using an anti-LinA antibody. Actin was used as a loading control. The full uncropped blot images for these figures are provided as Figure S2

Western blot analysis of LinA protein detected an approximately 18-kDa protein in four A5-derived plants (Fig. 3A), but not in wild type *Arabidopsis* plant (WT) or transgenic *Arabidopsis* E11-1 line transformed by pIG-sGFP [32]. The protein's molecular mass was larger than the 16.5-kDa molecular mass of native LinA expressed in bacterial strain UT26 [20]. In the previous study, LinA expressed in transgenic hairy root cultures of *C. moschata* was modified with high mannose-type* N*-linked oligosaccharide(s) [18]. *N*-glycosidase treatment using Endo H or PNGase F sifted the molecular mass to that of the native LinA expressed in bacterial cells (Fig. 3B). In contrast, the *O*-glycosidase treatment had no detectable effect (Fig. 3B), indicating that LinA in *Arabidopsis* was also modified with high mannose-type* N*-linked oligosaccharide(s). In addition, another band for a ca. 17-kDa protein was detected in this experiment and remained after *N*-glycosidase treatment (Fig. 3B), suggesting an additional unknown modification of LinA in *Arabidopsis.* A significantly higher amount of LinA was detected in the roots than in the leaves (Fig. 3C), indicating that LinA is accumulated more in the roots than in the leaves.

### Tolerance of transgenic *Arabidopsis* expressing LinA to γ-HCH

**Fig. 4:**
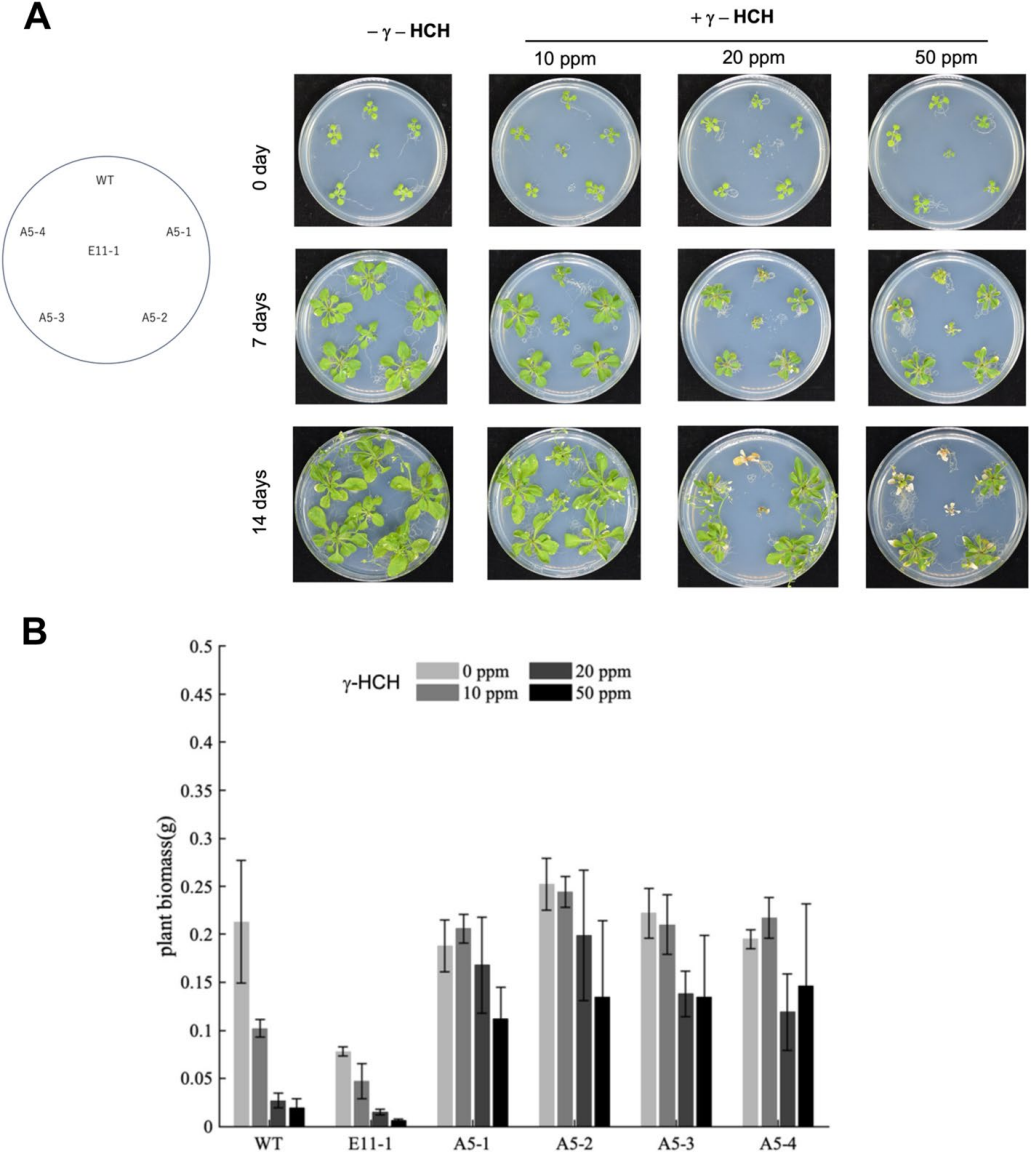
γ-HCH tolerance of transgenic *Arabidopsis* expressing LinA. Effects of γ-HCH on T3 transgenic *Arabidopsis* lines transformed with pAOs::relinA (A5-1 to A5-4) and with control empty vector (E11-1), and wild type *Arabidopsis* (WT) were estimated. **A** The seedlings of plants were transferred to MS medium containing γ-HCH at three different concentrations (10, 20, and 50 ppm) (0 day), and cultivated for 14 days. **B** The wet weight of plants cultivated on the medium containing γ-HCH for 14 days was measured. Error bars indicate the mean ± standard deviation (SD), *n* = 3 (biological replicates)

To estimate the effect of γ-HCH, the WT, E11-1, and A5-derived lines (A5-1 to A5-4) were cultivated on MS medium containing γ-HCH at three different concentrations (10, 20, and 50 ppm) (Fig. 4). No growth difference was observed in the 10 ppm γ-HCH condition (Fig. 4A). At 20 ppm, however, apparent growth defects were observed in WT and E11-1 but not in the A5-derived lines (Fig. 4A). A5-derived lines grew even under 50 ppm γ-HCH, although growth defects were observed to some extent (Fig. 4A). To quantitatively estimate the effects of γ-HCH on the plants, the fresh weights of plants grown on medium containing γ-HCH were measured (Fig. 4B). This result confirmed that *Arabidopsis* plants are sensitive to γ-HCH but becomes more tolerant to it by the expression of LinA.

### Degradation of γ-HCH by crude extracts of *Arabidopsis* expressing LinA

**Fig. 5:**
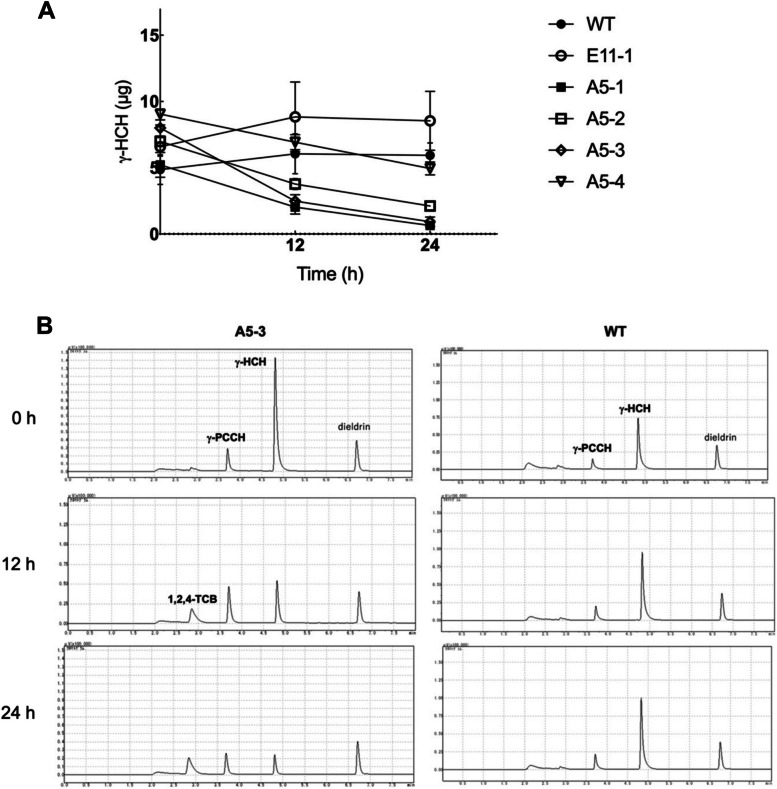
Degradation of γ-HCH by crude extracts of *Arabidopsis* expressing LinA. **A** Crude extracts of T3 transgenic *Arabidopsis* lines transformed with pAOs::relinA (A5-1 to A5-4) and with control empty vector (E11-1), and wild type *Arabidopsis* (WT) were added to the reaction buffer containing 10 ppm of γ-HCH, and the residual amounts of γ-HCH were measured by GC-ECD. Error bars indicate the mean ± standard deviation (SD), *n* = 3 (biological replicates). **B** Representative chromatograms of GC-ECD analysis for γ-HCH degradation by crude extracts of WT (right) and A5-3 line (left) plants. Chromatograms immediately after adding crude extract into the reaction buffer (0 h) and atter incubation for 12 and 24 h are shown. Dieldrin was added as the internal standard. Note that commercially available γ-HCH chemical contains γ-PCCH, and thus γ-PCCH was also detected in a negative control sample without crude extract

We prepared the crude extracts of WT, E11-1, and A5-derived lines, incubated each with 10 ppm of γ-HCH, and analyzed by gas chromatography with an electron capture detector (GC-ECD). Apparent decreases in γ-HCH were observed in the A5-derived lines but not in WT and E11-1 (Fig. 5A). The activity of A5-4 was slightly weaker than those of other A5- derived lines. Furthermore, the increase and decrease in γ-PCCH during the reaction and the appearance of 1,2,4-TCB were observed only in A5-devived lines. Representative chromatograms for WT and A5-3 are shown in Fig. 5B. These results indicated that LinA proteins expressed in A5-derived lines had γ-HCH dehydrochlorinase activity.

### Degradation of γ-HCH in liquid medium by the whole *Arabidopsis* plants expressing LinA

**Fig. 6:**
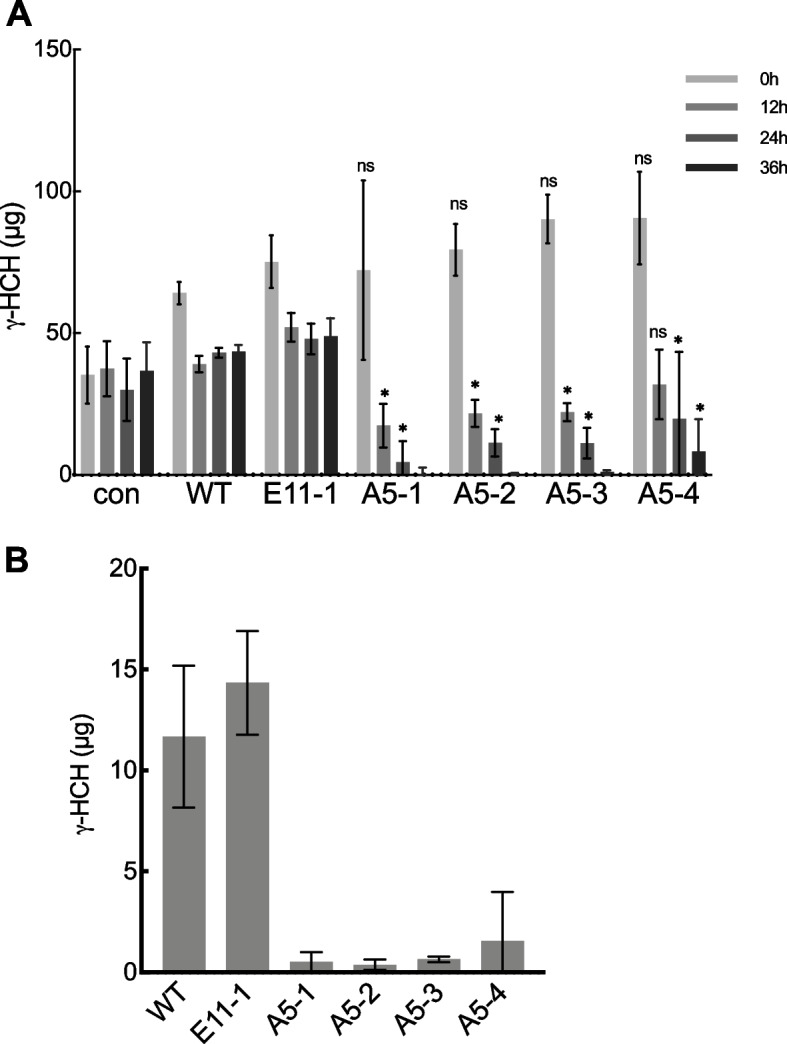
Degradation of γ-HCH in liquid medium by whole *Arabidopsis* expressing LinA. **A** Whole plants of T3 transgenic *Arabidopsis* lines transformed with pAOs::relinA (A5-1 to A5-4) and with control empty vector (E11-1), and wild type *Arabidopsis* (WT) were incubated in a liquid medium containing ten ppm of γ-HCH for 36 h. The concentration of γ-HCH in the medium was measured by GC-ECD, and total amount of γ-HCH in the medium was estimated. Error bars indicate the mean ± standard deviation (SD), *n* = 3 (biological replicates). Asterisks indicate significant differences from the WT detected by follow-up Two Factor ANOVA (*, *P* < 0.05). con, control without plant. **B** Total amount of γ-HCH in each plant body after incubation for 36 h was estimated. Error bars indicate the mean ± standard deviation (SD), *n* = 3 (biological replicates)

To assess the whole plant γ-HCH degradation activity of the A5-derived lines, the WT, E11-1, and A5-derived lines grown on solid medium were incubated in a liquid medium containing 10 ppm of γ-HCH. When whole plants of the A5-derived lines (A5-1, A5-2, and A5-3) were incubated in the medium, most of the γ-HCH in the medium disappeared after 36 h of incubation, unlike the case when whole plants of the WT and E11-1 lines or no plant were incubated in the medium (Fig. 6A). When whole plant of the A5-4 line was incubated in the medium, most of the γ-HCH in the medium disappeared after 36 h incubation, but a small amount of γ-HCH remained (Fig. 6A). In addition, far smaller amounts of γ-HCH were detected in the A5-derived line bodies after incubation in the medium for 36 h than in the WT and E11-1 line bodies (Fig. 6B). We estimated the total amount of γ-HCH in the assay system (Table 1). More than 99% of γ-HCH in the assay system disappeared when the A5-1, A5-2, and A5-3 lines were incubated in the medium. Despite a slightly lower reduction rate compared to the others, the A5-4 line caused more than 92% of γ-HCH in the assay system to disappear. These results indicated that the A5-derived lines can absorb and degrade γ-HCH in liquid medium.

**Table 1:** Change of total amount of γ -HCH in the assay system

		WT	E11-1	A5-1	A5-2	A5-3	A5-4
		Amounts of γ -HCH (µg)					
Before incubation	Medium	64.2 ± 1,2	75.2 ± 6.6	72.2 ± 29	79.4 ± 6.3	90.2 ± 5.8	90.6 ± 14
After 36 h incubation	Medium + Plant body	55.2 ± 4	63.4 ± 6.1	0.5 ± 0.42	0.4 ± 0.2	0.7 ± 0.1	7.1 ± 5.9
Reduction of γ -HCH(%)		14	15.7	99.3	99.5	99.3	92.2

Values are the mean ± standard deviation (SD), *n* = 3 (biological replicates)

## Discussion

In the present study, we generated the transgenic *Arabidopsis* into which the bacterial gene encoding γ-HCH dehydrochlorinase LinA was introduced. One of the T2 generation lines (A5) expressed and accumulated LinA protein well, and the A5-derived T3 lines degraded γ-HCH rapidly in liquid medium. This study is the first report on whole transgenic plants expressing active LinA. The transgenic *Nicotiana tabacum* plant expressing human cytochrome P4502E1 was reported to be capable of degrading γ-HCH [33]. However, the γ-HCH degradation activity of the transgenic *N. tabacum* decreased quickly. One of the reasons for this may be that P4502E1 requires a cofactor, NADPH, for its reaction. It is noteworthy that LinA did not require any cofactor for its reaction [20]. Thus, transgenic plants expressing LinA are more suitable for the sustainable removal of γ-HCH from the environment.

LinA converts γ-HCH into 1,2,4-TCB. Because of the high volatility of 1,2,4-TCB, it is plausible that 1,2,4-TCB evaporates during the incubation of γ-HCH with transgenic plants expressing LinA. Indeed, we did not detect 1,2,4-TCB in the γ-HCH degradation experiment in liquid medium. Since 1,2,4-TCB is toxic to plants [34, 35], it is advantageous that 1,2,4-TCB does not remain in the plant for continuous γ-HCH degradation. Moreover, the toxicity of 1,2,4-TCB for rats is approximately 10 times lower than that of γ-HCH [36, 37]. Therefore, the conversion of γ-HCH to 1,2,4-TCB at least reduces environmental toxicity. However, the possibility remains that 1,4-TCDN and 1,2,4-TCB are converted to other substances in plant cells by unknown enzymatic or nonenzymatic reaction(s). Further research on the metabolic pathway of γ-HCH in plant cells and the toxicological properties of the metabolites would be required for practical use of transgenic plants expressing LinA. LinA cannot degrade β-HCH [20, 22, 23], but LinB degrades β-HCH. Especially, a variant of LinB (LinB_MI_) from *Sphingobium* sp. strain MI1205 degrades β-HCH more effectively compared with LinB_UT_ from strain UT26 [38]. Co-expressing other *lin* genes, such as *linB* and *linC* [4], may be more efficient for the complete detoxification of an HCH-contaminated site.

Although we obtained six T2 lines into which *relinA* was introduced (Fig. 2A), only one plant line (A5) expressed *relinA* well at both transcriptional (Fig. 2B) and translational (Fig. 3A) levels. The genome positions into which the *relinA* gene was integrated and the copy number of the introduced *relinA* gene may be important for the stable expression of LinA in plant cells. Even among the T3 A5-derived lines, A5-4 showed weaker LinA activity than the other three A5-derived lines (Figs. 4, 5 and 6). Although the reason remains unclear, selecting a stable line that expresses LinA well among many transgenic plant lines is crucial for the practical phytoremediation.

LinA was modified by glycosylation with high mannose-type *N*-linked oligosaccharide(s) in the hairy roots culture of *C. moschata* [18]. LinA expressed in *A. thaliana* also underwent the same glycosylation, although LinA expressed in *Arabidopsis* may have undergone additional modification(s) (Fig. 3B)*.* The glycosylation probably does not significantly affect LinA activity since the predicted glycosylation residue N137 is not an amino acid residue critical to the LinA reaction [18]. Indeed, LinA expressed in *Arabidopsis* showed γ-HCH dehydrochlorinase activity without glycosidase treatment. Glycosylation may be important for the stable accumulation of LinA in plant cells.

WT *Arabidopsis* and the E11-1 line were sensitive to γ-HCH at more than 20 ppm of γ-HCH on the solid medium (Fig. 4). Since the A5-derived lines expressing active LinA showed tolerance to γ-HCH, γ-HCH itself seems toxic towards the *Arabidopsis* plant. The phytotoxic effects of γ-HCH on other plants have also been reported [33, 39]. γ-HCH causes nerve excitation symptoms by blocking an insect's central nervous system GABA (γ-aminobutyric acid) receptor [40]. However, the effects of γ-HCH on plants varied depending on the species [39], and the toxic mechanism of γ-HCH in plants remains unknown.

In a preliminary experiment, we assessed the ability of the A5-derived lines to degrade γ-HCH in soil. Although no apparent reduction of γ-HCH in soil was observed, the amounts of γ-HCH in plant bodies of the A5-derived lines were lesser than that of WT plants after cultivation in γ-HCH-polluted soil, suggesting that γ-HCH absorbed from soil was degraded in the plant bodies of the A5-derived lines (data not shown). LinA expressed in the transgenic *Arabidopsis* plant accumulated more in the roots than in the leaves (Fig. 3C). Although the reason for this remains unclear, this feature benefits soil's phytoremediation of γ-HCH. In any case, absorption of γ-HCH from soil is crucial for the practical use of transgenic plants. Therefore, generating whole transgenic *Cucurbita* species expressing LinA is a reasonable strategy for phytoremediation of γ-HCH in soil, as suggested previously [18].

## Conclusion

The successful establishment of whole transgenic *Arabidopsis* plants expressing and accumulating active LinA is a crucial step toward the practical use of transgenic plants in the phytoremediation of γ-HCH. We demonstrated that these plants absorbed and degraded γ-HCH in a liquid medium. This study confirms the possibility of a new strategy for the use of transgenic plants for the degradation of γ-HCH in the environment. We believe that the present results will play a vital role for the practical application of transgenic plants to remediate POPs.

## Methods

### Plant materials and growth conditions

Seeds of *Arabidopsis thaliana* (Columbia ecotype) were surface-sterilized with 20% NaClO for 15 min, followed by washing five times with sterile water. The sterilized *Arabidopsis* seeds were put on Murashige and Skoog (MS) medium with 0.8% agar in Petri dish. After incubation at 4°C for 72 h in darkness, the Petri dish was transferred into a growth incubator for germination and development (22°C, 16 h light/8 h dark). After incubation for 7 days, the seedlings were transplanted into soil and grew in a growth chamber (22°C, 16 h light/8 h dark).

### Construction of transgenic *Arabidopsis* plants

The vector pAOs::relinA [18] was introduced into *Agrobacterium tumefaciens* strain EHA105 by electroporation [41]. Using the resultant strain, *A. thaliana* plants were transformed by floral dip method [42]. Seeds of T0 transgenic plants were screened on MS medium containing 50 mg/L of kanamycin and carbenicillin 100 mg/L, after which the T1 plant seedlings were transferred to soil. Finally, the homozygous genotypes of T2 transgenic plants were obtained from self-fertilization, and homozygous lines were identified in the T3 generation via segregation analysis. One of the T3 transgenic *Arabidopsis* lines (E11-1) transformed by pIG-sGFP [32] was used as a negative control (empty) line.

### DNA extraction from *Arabidopsis* and PCR analysis

Genomic DNA of *Arabidopsis* was extracted from the leaves of 14- to18-day-old plants as described previously [43]. PCR analysis was performed using a primer set (*AOs*::*relinA*, 5′- CCTAGAAGCTAATTCCCGATCTAG-3′ and 5′- AAGGCCATCGTTGAAG-3′) as follow: 94 °C for 2 min, 30 cycles at 94 °C for 30 s, 52 °C for 30 s, and at 68 °C for 150 s, and a final extension step at 68 °C for 5 min. PCR products were analyzed by electrophoresis using 1.0% agarose gel.

### RNA extraction from *Arabidopsis* and RT-qPCR analysis

Total RNA of *Arabidopsis* was extracted from 14-day-old plants by RNeasy Plant Mini Kit (Qiagen) and treated with DNase I (Takara) at 37 °C for 1 h to eliminate contaminated genomic DNA. The total RNA was reverse transcribed using the SuperScript II First-Strand Sythesis System (Thermo Fisher Scientific) with an Oligo dot Primer. Real-time DNA amplification using primer sets (*relinA*, 5′-CGCTGACAAAGTGAACGGTA-3′ and 5′-TAGTTCGTGCATGCATTCCT-3′; *ACTIN2*, 5′-GCACCACCTGAAAGGAAGTACA-3′ and 5′-CGATTCCTGGACCTGCCTCATC-3) was monitored using Bio-Rad CFX Maestro software (Bio-Rad Laboratories). The expression level of the *relinA* gene was normalized to that of the endogenous reference gene *ACTIN2*.

### Preparation of crude extract from *Arabidopsis* and bacterial cells

Leaves and roots of 14- to 18-day-old *Arabidopsis* were ground using a disposable pestle (Bio-Bik), and their total soluble proteins were extracted with an extraction buffer containing 50 mM HEPES–KOH (pH7.5), 100 mM NaCl, 2.5 mM EDTA, 1 mM PMSF, 20 µM Leupeptin, and 1 mM DTT. The homogenate was then centrifuged at 12,000 rpm at 4 °C for 2 min to remove debris. Total proteins were extracted from bacterial strain UT26 as follows: UT26 cells grown at 30 °C in 1/3LB (3.3 g of Bacto tryptone, 1.7 g of yeast extracts, and 5 g of sodium chloride per liter) were resuspended in 50 mM potassium phosphate buffer (pH 7.5), and disrupted by sonication (Branson Sonifier 250A). After centrifugation, the supernatant was used as crude extract. Protein concentration was estimated by Bradford method with bovine γ-globulin as a standard.

#### Western blot analysis

Protein samples were separated using SDS-PAGE with 15% gel and transferred to PVDF membranes (Trans-Blot Turbo Trans Pack, Bio-Rad Laboratories) using Trans-Blot Turbo System (Bio-Rad Laboratories). Membranes were blocked for 30 min in 5%(w/v) nonfat dry milk in TBS-T containing 25 mM Tris–HCl (pH7.4), 0.15 M NaCl, and 0.05%(v/v) Tween-20. The membranes were incubated with an anti-LinA antibody [44] (1: 2,000 dilution) or an anti-Actin (Plant) antibody (1:3,000 dilution, Sigma-Aldrich) in Solution I for primary antibody (Can Get Signal™ Immunoreaction Enhancer Solution, Toyobo) at 4 °C overnight, followed by incubation with anti-rabbit IgG HRP or anti-mouse IgG HPR (Thermo Fisher Scientific) in Solution II for secondary antibody (Can Get Signal™ Immunoreaction Enhancer Solution, Toyobo) at room temperature for 2 h. Antigen–antibody complexes were detected using an Chemi-Lumi Ultra (Nacalai Tesque).

#### LinA glycosylation analysis

The total soluble proteins were deglycosylated by treatment with Endo H, PNGase F and *O-*glycosidase (New England BioLabs) under denaturing conditions according to the manufacturer’s instructions. Western blot analysis was performed for the treaded samples.

#### γ-HCH tolerance assay using whole plants

Seeds of *Arabidopsis* were germinated on MS medium with 0.8% agar containing 50 mg/L of kanamycin and grew in an incubator for germination and development (22 °C, 16 h light/8 h dark). After cultivation for 14 days, the whole plants were transplanted to MS medium with 0.8% agar containing 10, 20 and 50 ppm of γ-HCH, and incubated for development (22 °C, 16 h light/8 h dark). Fresh weights of the whole plants after 14 days cultivation were measured.

#### γ-HCH degradation assay using crude extracts

Leaves and roots of 14- to 18-day-old *Arabidopsis* plants were immersed in liquid nitrogen and ground to fine powder using a mortar and pestle. The fine powder was mixed with extraction buffer containing 50 mM potassium phosphate buffer (pH 7.5) and centrifuged at 6,200 rpm at 4 °C for 10 min. The supernatant was used as the crude extract. In a 1 mL glass reaction vial with a mininat valve (Osaka Chemical, Osaka, Japan), 200 µL of the crude extract was mixed with 800 µL glycine–NaOH buffer (pH 8.6). After 1 µL of 10 mg/mL γ-HCH was added to the solution, the mixture was incubated at 30 °C. At appropriate time points, 100 µL was sampled and then mixed with an equivalent volume of ethyl acetate containing 2 ppm of dieldrin as the internal standard. After centrifugation, the ethyl acetate layer was recovered and analyzed by GC-ECD.

#### GC-ECD analysis

GC-ECD analysis was conducted using a GC-17A gas chromatograph (Shimadzu, Kyoto, Japan) and a Rtx-1 capillary column (30 m × 0.25 mm × 0.25 µm; Restek, Bellafonte, PA, USA). The column temperature was increased from 160 °C to 280 °C at a rate of 40 °C/min, and the gas flow rate was 30 mL/min. γ-HCH was purchased from Tokyo Chemical Industry Co. (Tokyo, Japan).

#### γ-HCH degradation assay using whole plants

Seeds of *Arabidopsis* plants were germinated in MS medium with 0.8% agar containing 50 mg/L of kanamycin and grew in a incubator for germination and development (22 °C, 16 h light/8 h dark). After cultivation for 14 days, the whole plants were transferred to 10 mL of MS liquid medium containing 10 ppm of γ-HCH (100 µg of γ-HCH in the assay system), and incubated at 22 °C on a rotary shaker (100 rpm) for 36 h. At appropriate time points, 100 µL of the medium was sampled and then mixed with an equivalent volume of ethyl acetate containing 2 ppm of dieldrin as the internal standard. The ethyl acetate layer was recovered after centrifugation and analyzed by GC-ECD. After incubation for 36 h, whole plant was immersed in liquid nitrogen and ground to fine powder using a mortar and pestle. The fine powder was mixed with extraction buffer containing 50 mM potassium phosphate buffer (pH 7.5) and then mixed with an equivalent volume of ethyl acetate containing 2 ppm of dieldrin as the internal standard. After centrifugation, the ethyl acetate layer was recovered and analyzed by GC-ECD.

### Supplementary Information

Supplementary Material 1.

## Data Availability

The data that support the findings of this study are available from the corresponding author upon reasonable request.
