# Supplementary material for: Transgenic Arabidopsis thaliana plants expressing bacterial γ-hexachlorocyclohexane dehydrochlorinase LinA
Source: BMC Biotechnol. 2024 Jun 19;24:42. doi: 10.1186/s12896-024-00867-0 (PMC11186250; doi:10.1186/s12896-024-00867-0)
Supplement: Supplementary file 1 — Supplementary Material 1. [file 12896_2024_867_MOESM1_ESM.zip › BMC Biotech_Deng_Sup_240304.pdf]

SUPPLEMENTARY MATERIAL

Transgenic *Arabidopsis thaliana* plants expressing bacterial γ-hexachlorocyclohexane dehydrochlorinase LinA

Wenhao Deng[1], Yoshinobu Takada[1], Yoshihiko Nanasato[2], Kouhei Kishida[1], Leonardo Stari[1], Yoshiyuki Ohtsubo[1], Yutaka Tabei[3], Masao Watanabe[1], and Yuji Nagata[1,*]

[1] Department of Molecular and Chemical Life Sciences, Graduate School of Life Sciences, Tohoku University, Sendai 980-8577, Japan

[2] Forest Bio-Research Center, Forestry and Forest Products Research Institute (FFPRI), Forest Research and Management Organization (FRMO), 3809-1 Ishi, Juo, Hitachi, Ibaraki 319-1301, Japan

[3] Faculty of Food and Nutritional Sciences, Toyo University, 1-1-1 Izumino, Itakura-machi, Ora-gun, Gunma 374-0193, Japan,

This PDF file includes:

Figure S1: The full uncropped gel images for Figure 2A

Figure S2: The full uncropped blot images for Figure 3

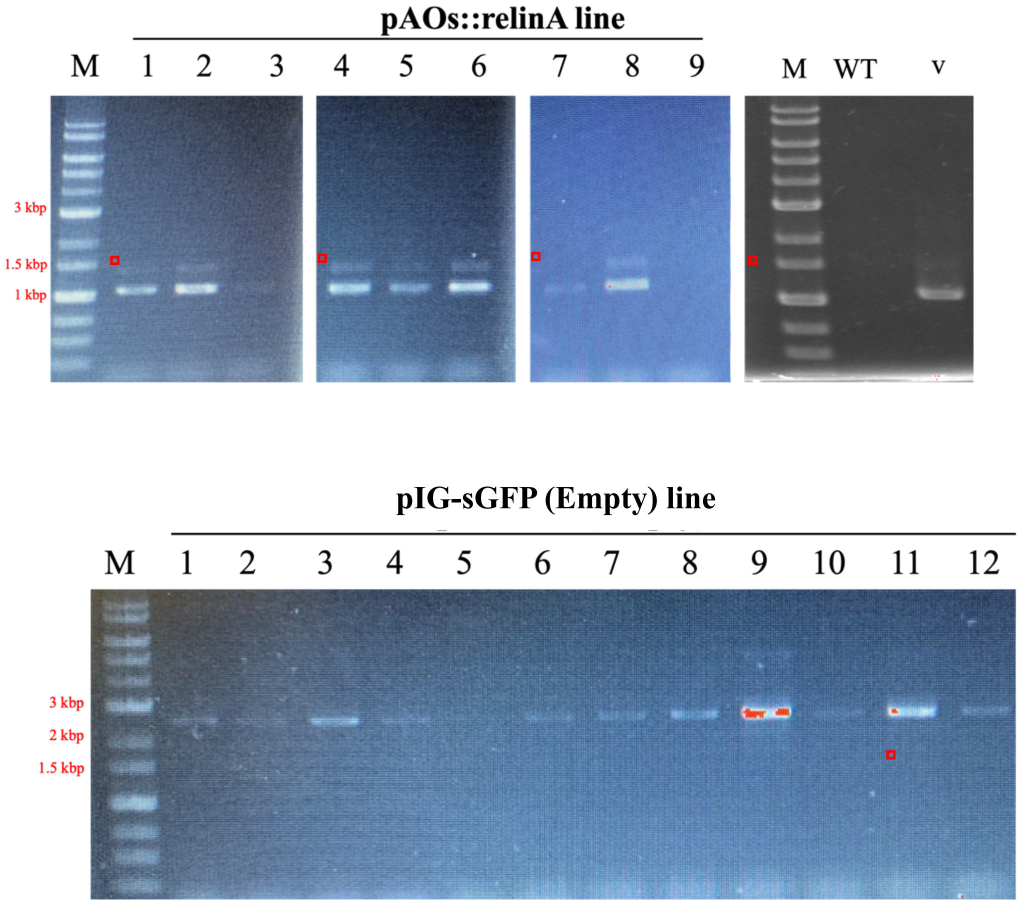

Figure S1. The full uncropped gel images for Figure 2A

The rectangles bordered in red were used to create Figure 2A. The pIG-sGFP plasmid has the *gfp* gene in the place of the *relinA* gene in pAOs::relinA. Thus, theoretically, 2,598 bp DNA is amplified by the primer set for the *relinA* gene. The original gel images for these figures are available as tiff files (Deng_Figure_S1_01 to Deng_Figure_S1_05)

A

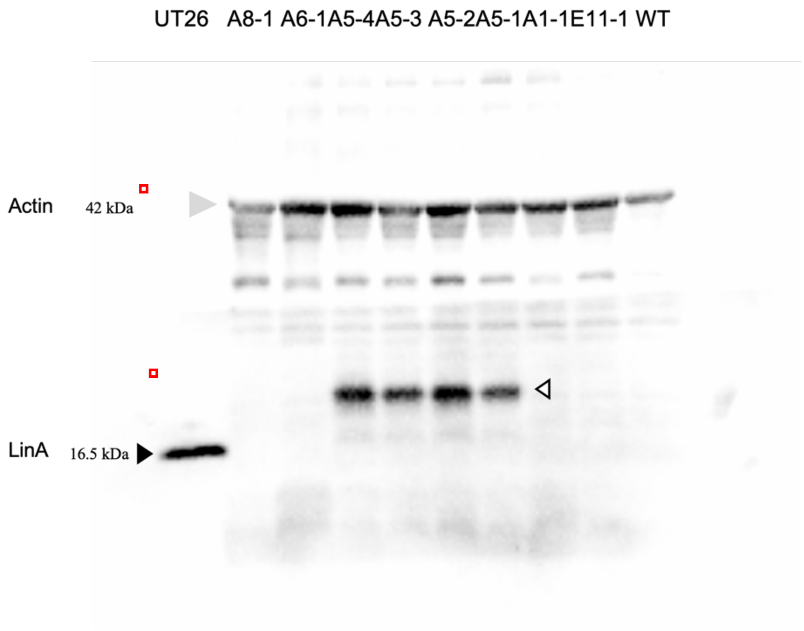

UT26 A8-1 A6-1A5-4A5-3 A5-2A5-1A1-1E11-1 WT
Actin
42 kDa
LinA
16.5 kDa

B

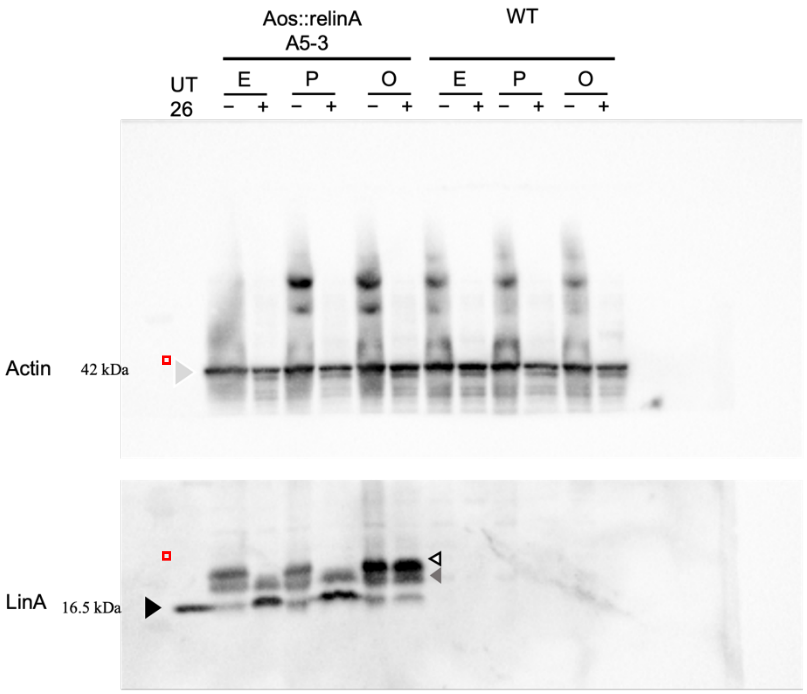

Aos::relinA
A5-3
WT
UT
26
E
P
O
E
P
O
– + – + – + – + – + – +
Actin
42 kDa
LinA
16.5 kDa

C

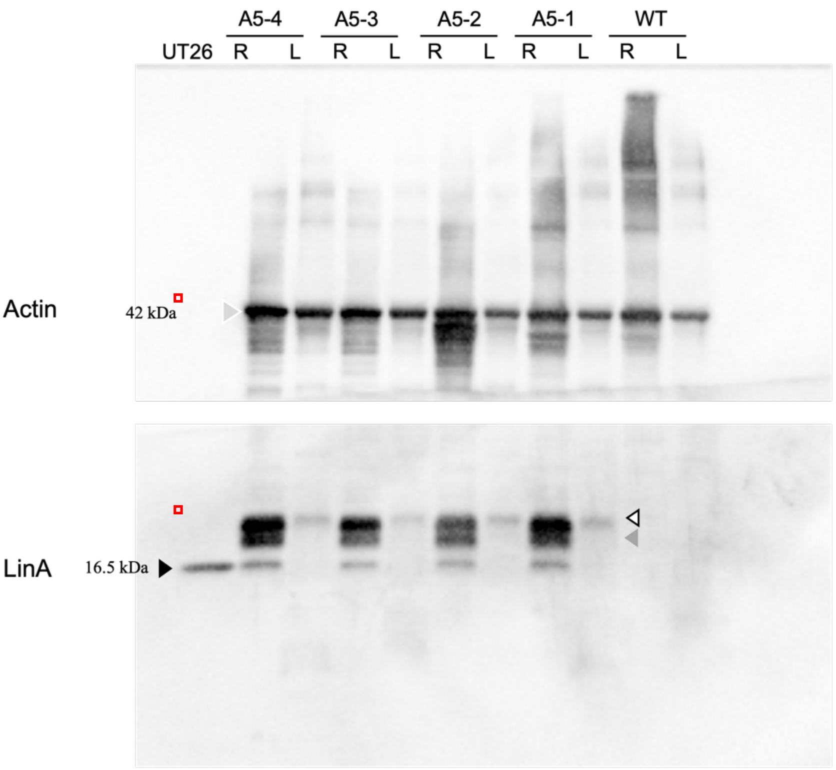

Figure S2. The full uncropped blot images for Figure 3

The rectangles bordered in red in panel A, B, and C were used to create Figure 3A, 3B, and 3C, respectively. The original blot images for these figures are available as tiff files (Deng_Figure_S2A_01, Deng_Figure_S2B_01, Deng_Figure_S2B_02, Deng_Figure_S2C_01, and Deng_Figure_S2C_02)
