# Supplementary figures and images for: Transgenic Arabidopsis thaliana plants expressing bacterial γ-hexachlorocyclohexane dehydrochlorinase LinA
Source: BMC Biotechnol. 2024 Jun 19;24:42. doi: 10.1186/s12896-024-00867-0 (PMC11186250; doi:10.1186/s12896-024-00867-0)

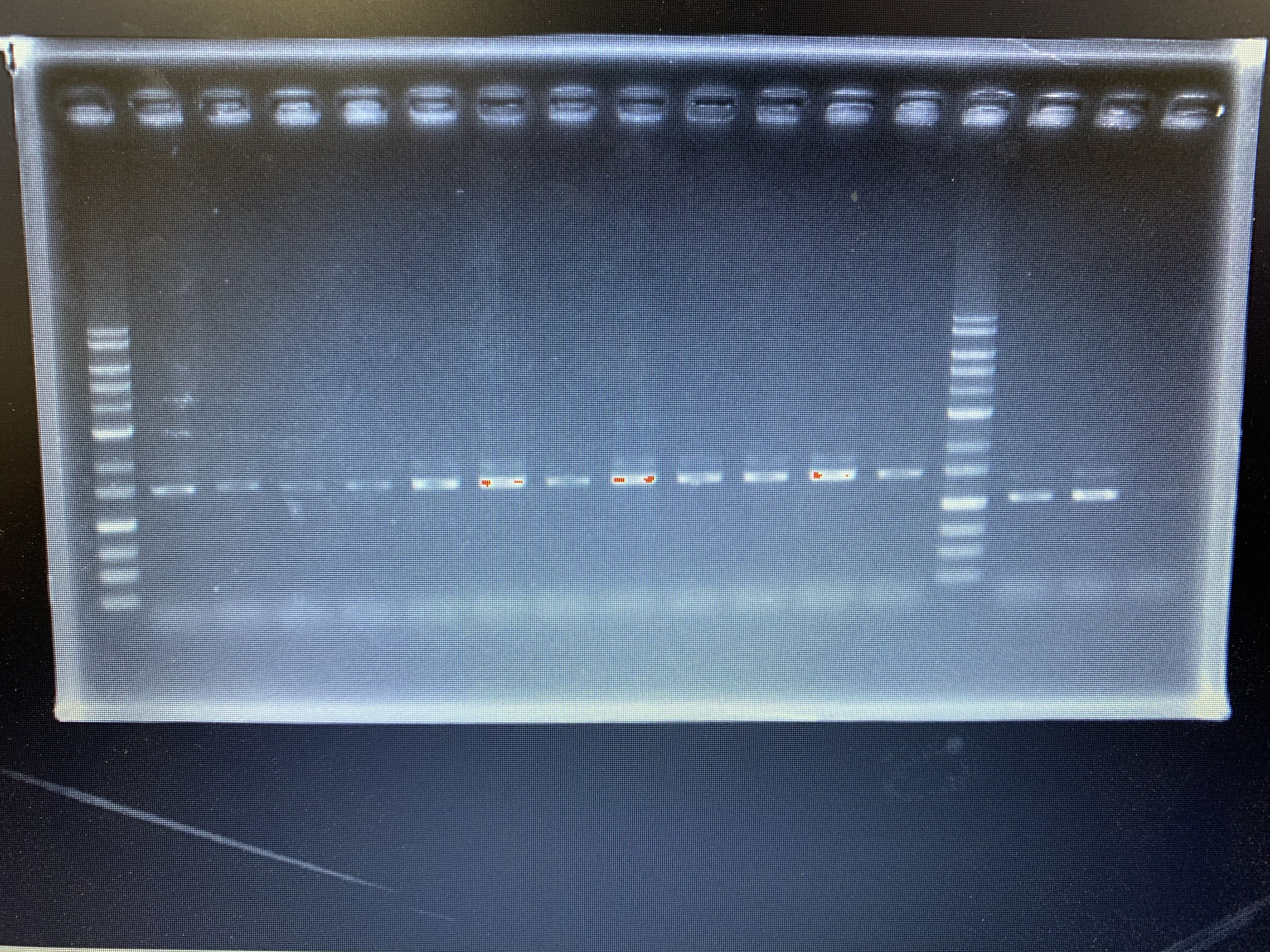

Supplement: Supplementary file 1 — Supplementary Material 1. [file 12896_2024_867_MOESM1_ESM.zip › Deng_Figure_S1_01.tiff]

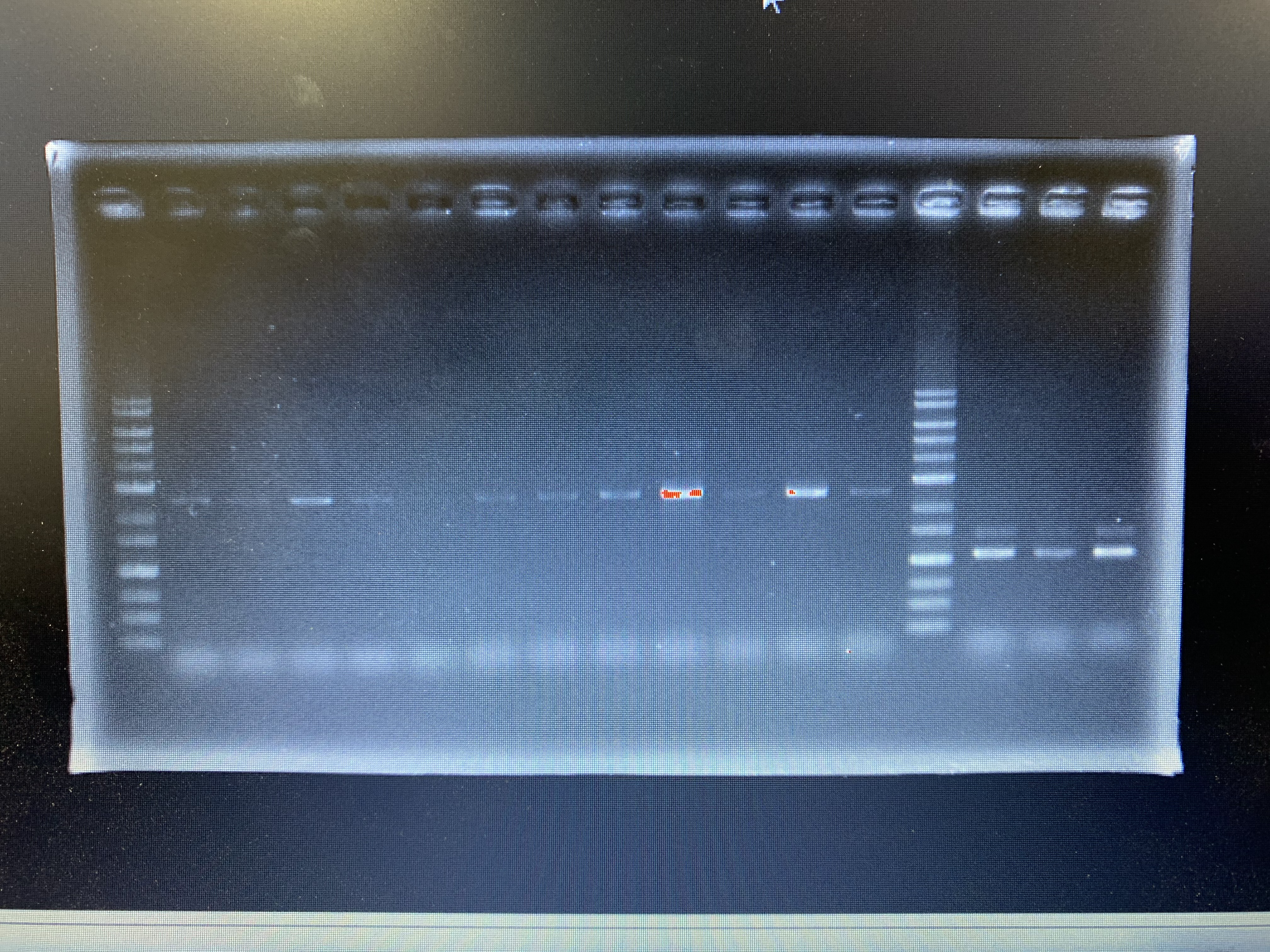

Supplement: Supplementary file 1 — Supplementary Material 1. [file 12896_2024_867_MOESM1_ESM.zip › Deng_Figure_S1_02.tiff]

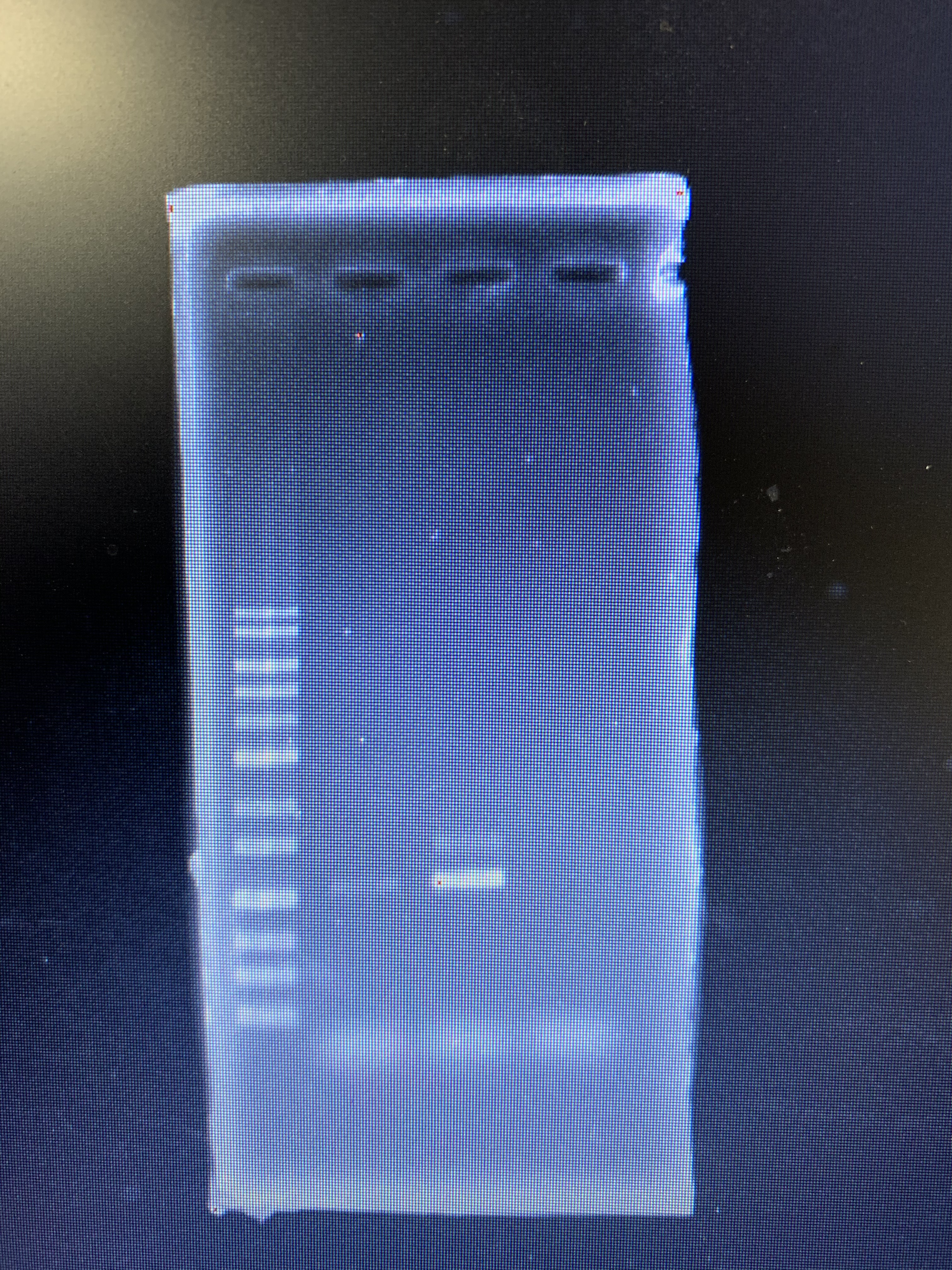

Supplement: Supplementary file 1 — Supplementary Material 1. [file 12896_2024_867_MOESM1_ESM.zip › Deng_Figure_S1_03.tiff]

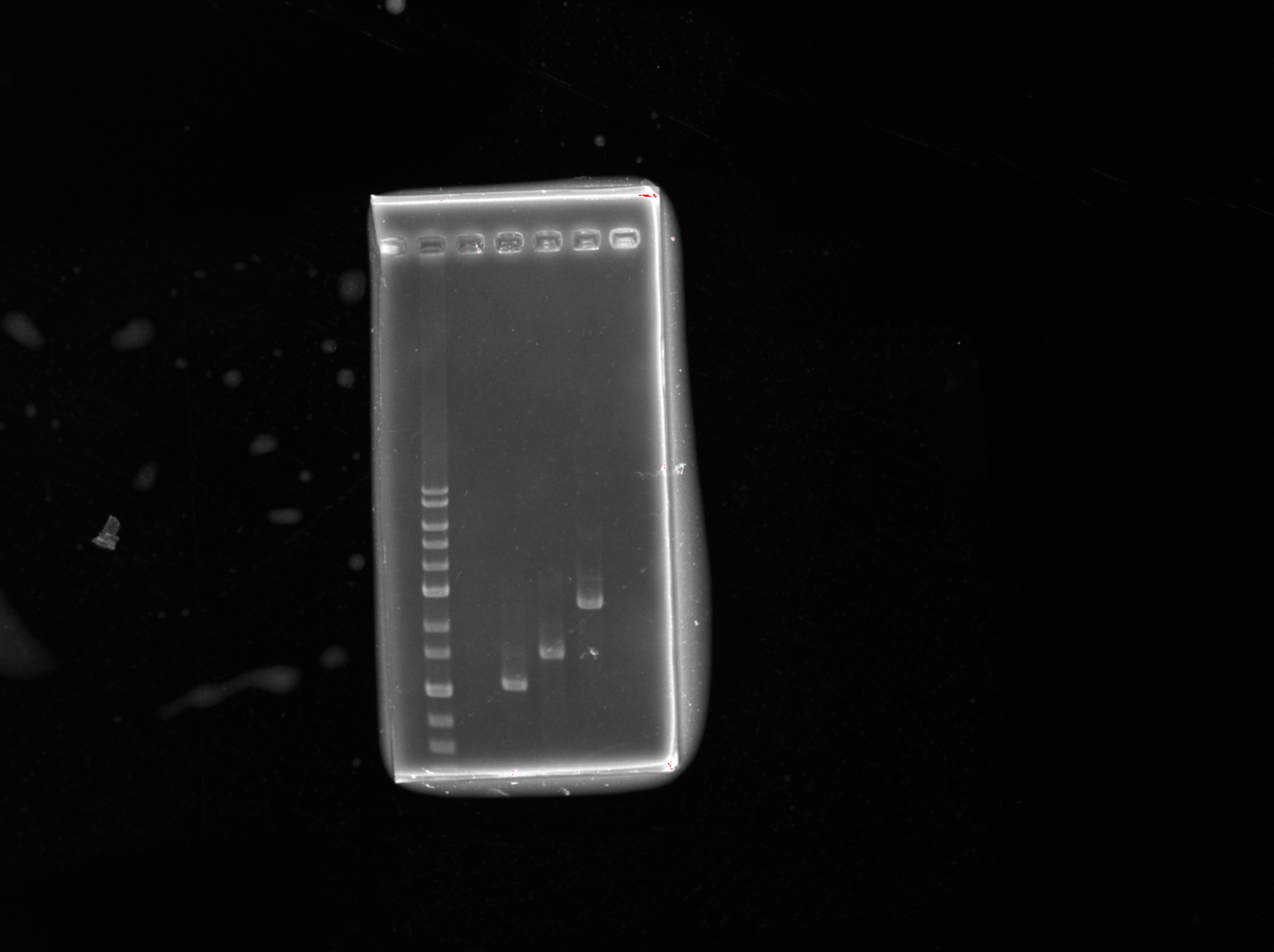

Supplement: Supplementary file 1 — Supplementary Material 1. [file 12896_2024_867_MOESM1_ESM.zip › Deng_Figure_S1_04.tif]

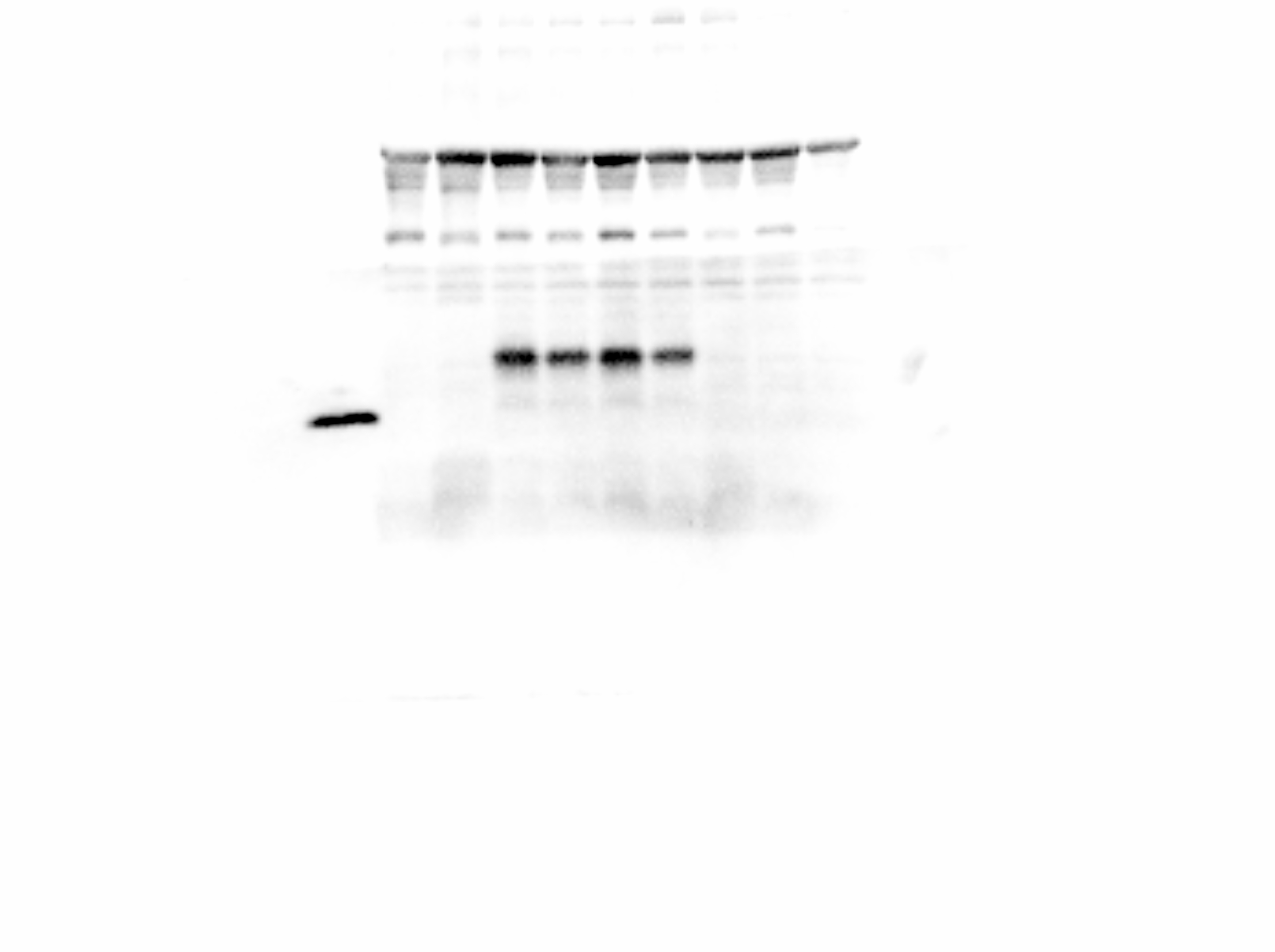

Supplement: Supplementary file 1 — Supplementary Material 1. [file 12896_2024_867_MOESM1_ESM.zip › Deng_Figure_S2A_01.tiff]

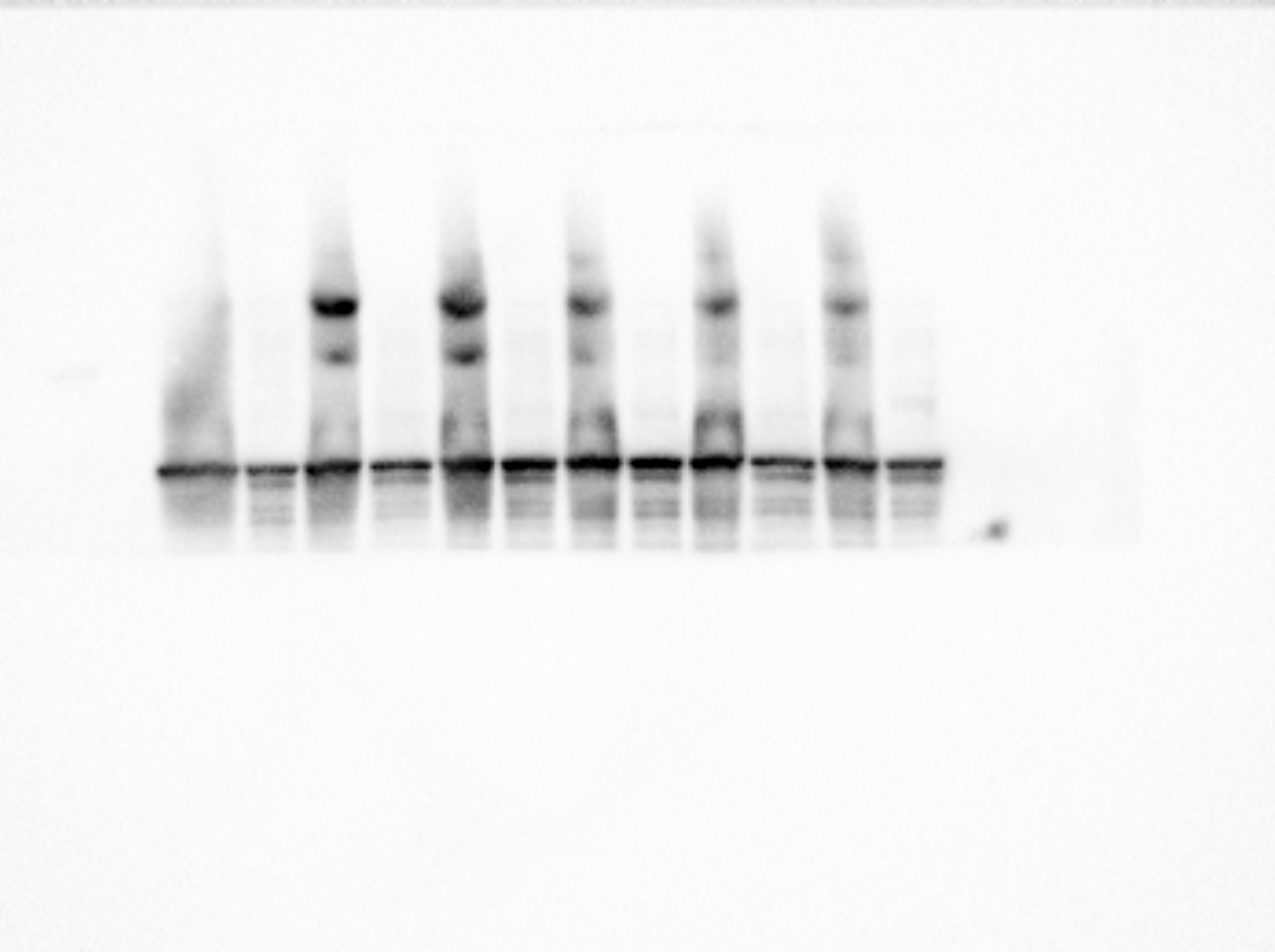

Supplement: Supplementary file 1 — Supplementary Material 1. [file 12896_2024_867_MOESM1_ESM.zip › Deng_Figure_S2B_01.tiff]

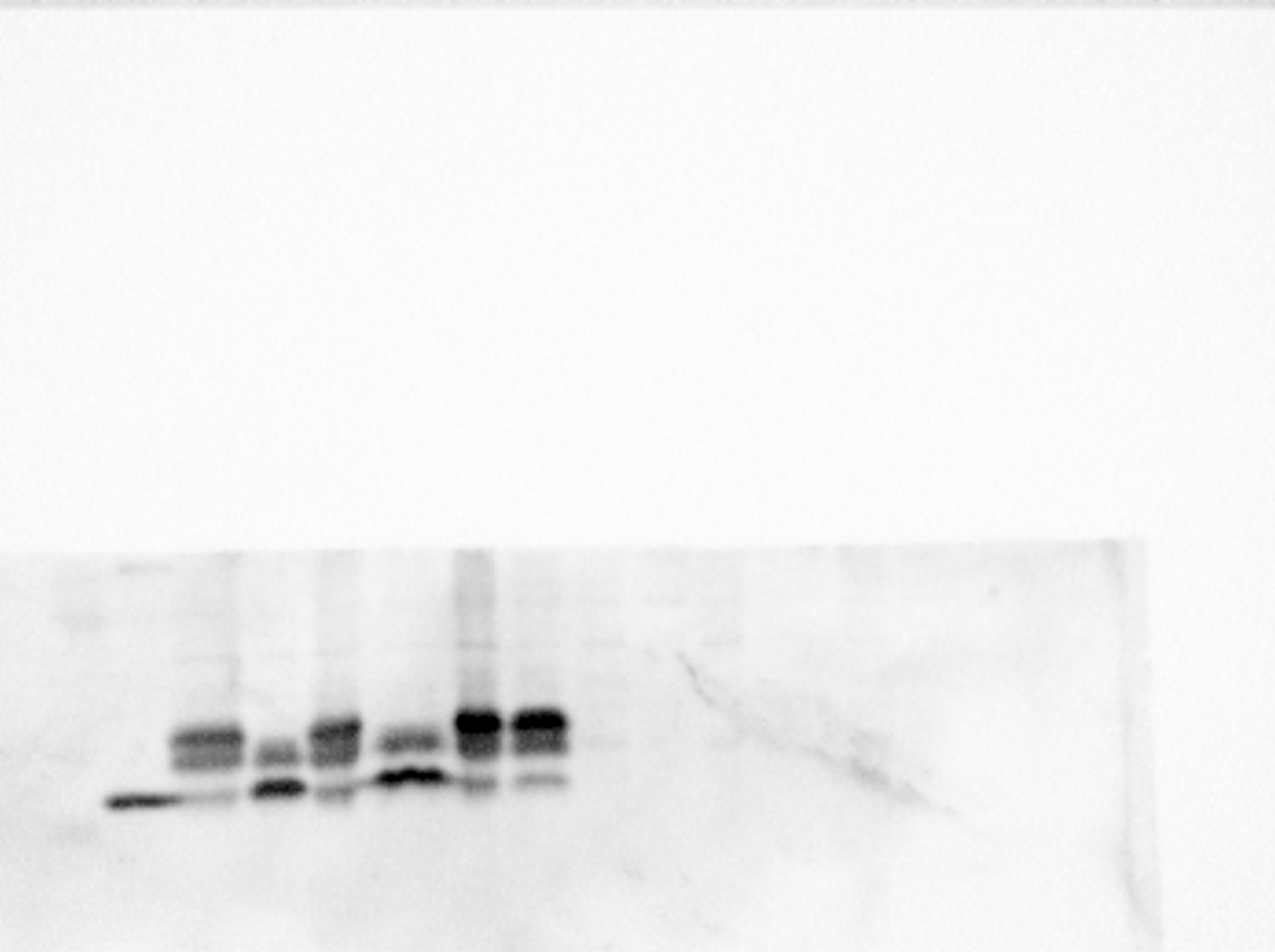

Supplement: Supplementary file 1 — Supplementary Material 1. [file 12896_2024_867_MOESM1_ESM.zip › Deng_Figure_S2B_02.tiff]

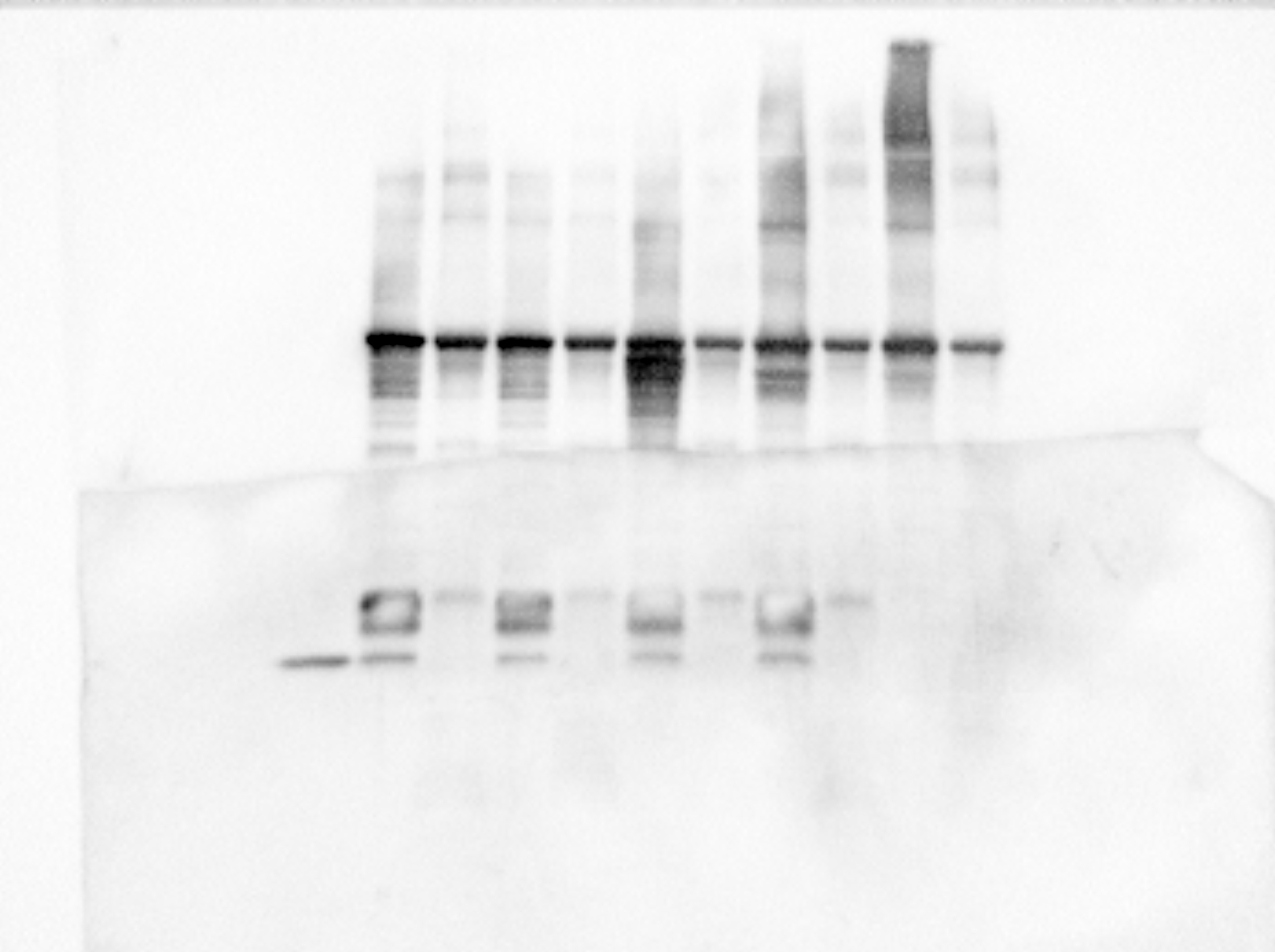

Supplement: Supplementary file 1 — Supplementary Material 1. [file 12896_2024_867_MOESM1_ESM.zip › Deng_Figure_S2C_01.tiff]

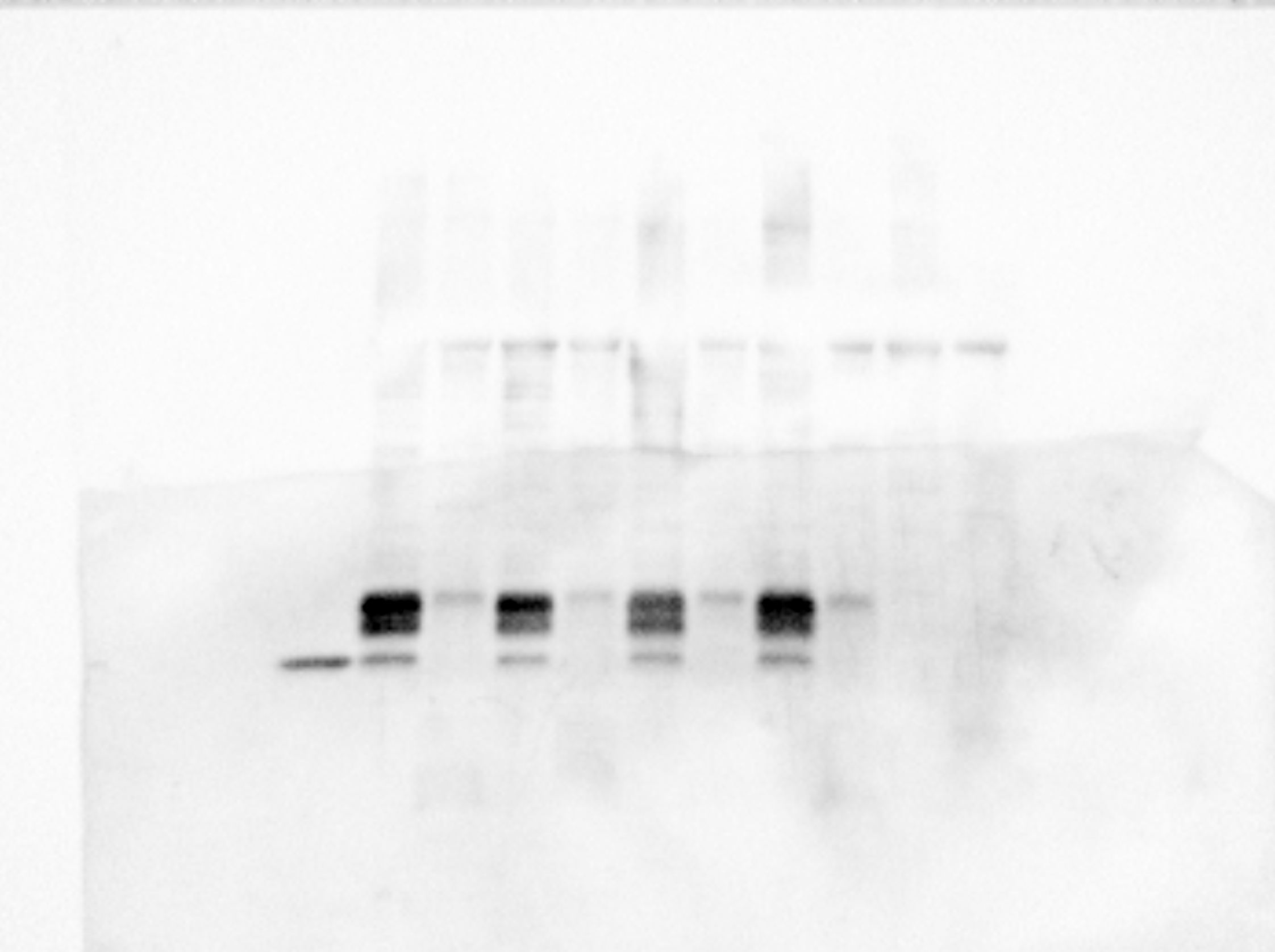

Supplement: Supplementary file 1 — Supplementary Material 1. [file 12896_2024_867_MOESM1_ESM.zip › Deng_Figure_S2C_02.tiff]
